# Herb-Induced Liver Injury: A Case of Acute Hepatotoxicity Associated with Horny Goat Weed Use

**DOI:** 10.51894/001c.144607

**Published:** 2025-09-30

**Authors:** Mohamed Shueb

**Affiliations:** 1 Department of Internal Medicine Detroit Medical Center-Sinai Grace Hospital

## 103

### INTRODUCTION

The widespread availability of over-the-counter herbal supplements has raised concerns about their potential health risks, particularly liver toxicity. This report discusses a case of acute liver injury potentially linked to prolonged use of Horny Goat Weed, an herbal supplement commonly used to enhance libido.

### CASE DESCRIPTION

A 53-year-old male presented to the emergency department with a one-day history of nausea, vomiting, dark urine, and yellowish discoloration in the eyes. He had reduced food intake due to nausea and vomiting but denied recent acetaminophen use, alcohol consumption, blood transfusions, fever, chills, or changes in bowel habits. The patient’s medical history was unremarkable, and there was no family history of liver disease. Notably, he had been using Horny Goat Weed daily for over a year. Upon admission, laboratory results revealed markedly elevated liver enzymes (Total bilirubin 4.94, Direct Bilirubin 4.33, ALT 516, AST 185, alkaline phosphatase 320, and GGT 357), which worsened, resulting in right upper quadrant pain and persistent nausea. Imaging, including a CT scan and right upper quadrant ultrasound, showed no abnormalities such as gallstones or tumors. An MRCP was not needed due to normal imaging. An EGD revealed mild gastritis, grade 1 varices, and early portal hypertension.

Further investigations ruled out autoimmune hepatitis, hemochromatosis, Wilson’s disease, and other liver disorders. Medications like atorvastatin and acetaminophen were discontinued. Liver function improved with supportive care. The patient was advised to avoid Horny Goat Weed, limit acetaminophen use, and refrain from restarting atorvastatin.

### DISCUSSION

Horny Goat Weed contains icariin, which may cause liver injury through oxidative stress or immune-related mechanisms. This case emphasizes the importance of detailed histories regarding herbal supplement use in patients with liver dysfunction. Timely cessation of suspected hepatotoxins, along with supportive care, is essential for recovery.

### CONCLUSION

This report highlights the liver toxicity risks associated with Horny Goat Weed. Healthcare providers should be aware of the hepatotoxic risks of herbal supplements and educate patients about potential adverse effects. Further research is needed to understand herb-induced liver injury better.

